# Supplementary figures and images for: Protein composition of axonal dopamine release sites in the striatum
Source: eLife. 2022 Dec 29;11:e83018. doi: 10.7554/eLife.83018 (PMC9937654; doi:10.7554/eLife.83018)

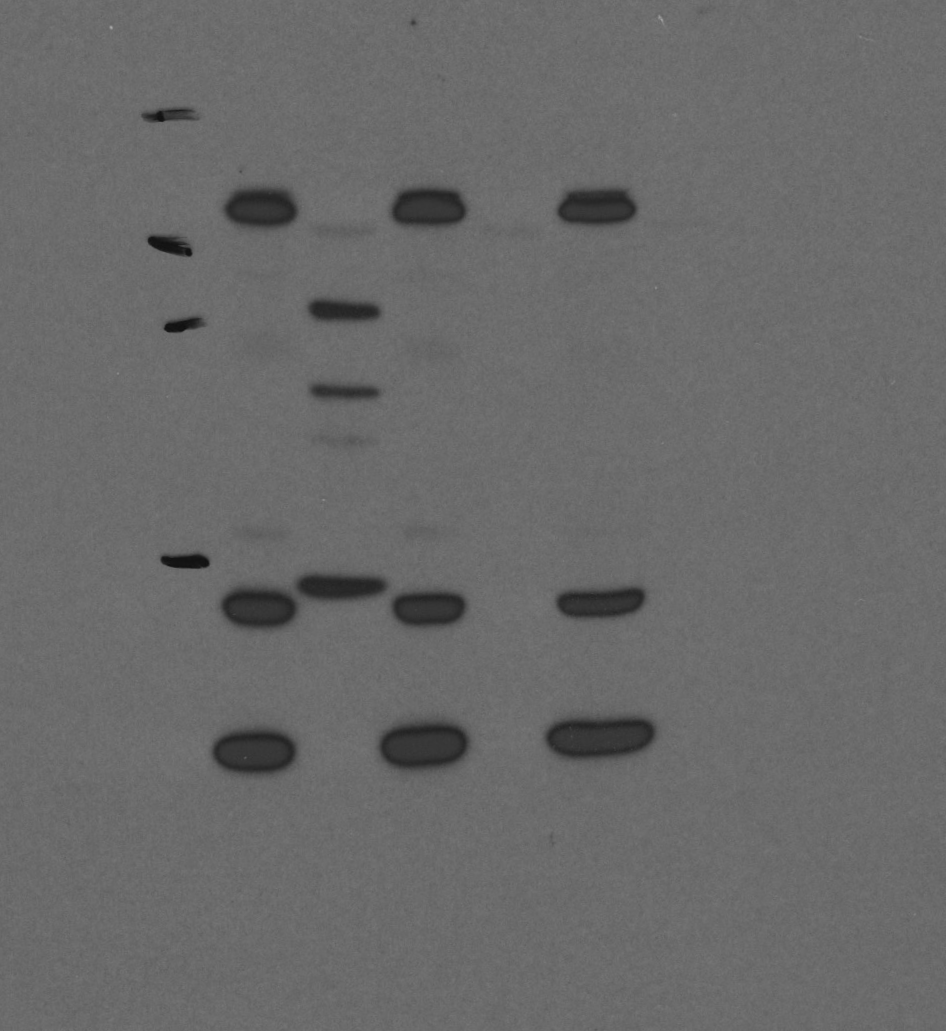

Supplement: Figure 1—figure supplement 2—source data 1. — (A) Original scan (5 min) of anti-RFP Western blots shown in Figure 1—figure supplement 2A. (B) Grayscale scans (left) and brightness and contrast-adjusted scans (right) of Western blots shown in Figure 1—figure supplement 2A. Stars denote bands that are likely degradation products of the BirA-tdTomato fusion protein. Arrows denote bands that are likely cross-reactive; they are present independent of BirA-tdTomato expression and are strong in intensity because a long exposure is shown and a large amount of input was loaded. (C) Original scan (3 s) of anti-β-actin Western blots shown in Figure 1—figure supplement 2A. (D) Grayscale scans (left) and brightness and contrast-adjusted scans (right) of Western blots shown in Figure 1—figure supplement 2A. Arrowheads denote bands that likely represent synapsin isoforms. [file elife-83018-fig1-figsupp2-data1.zip › SFigure1_S2_Source_Data_1A-v1.tif]

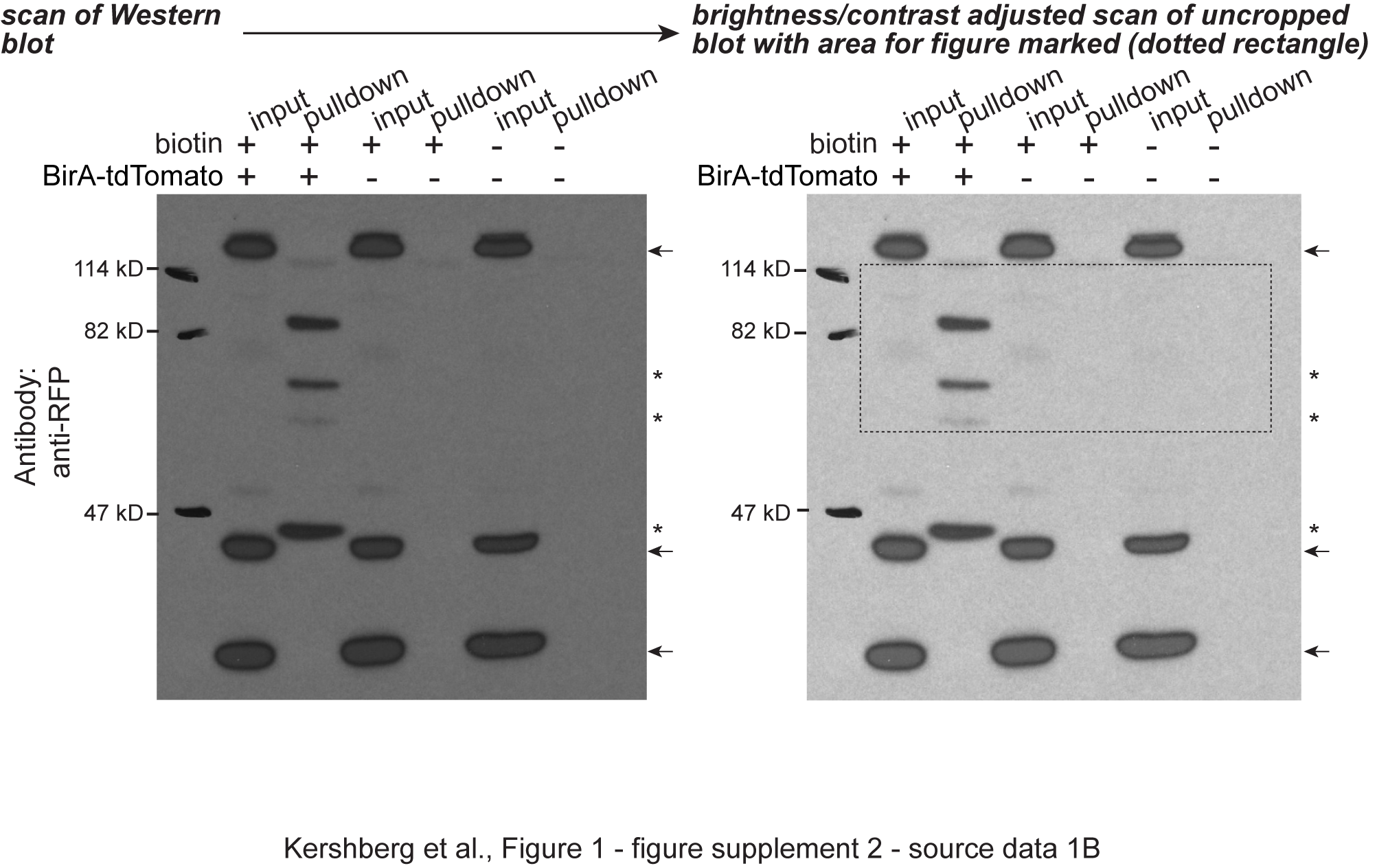

Supplement: Figure 1—figure supplement 2—source data 1. — (A) Original scan (5 min) of anti-RFP Western blots shown in Figure 1—figure supplement 2A. (B) Grayscale scans (left) and brightness and contrast-adjusted scans (right) of Western blots shown in Figure 1—figure supplement 2A. Stars denote bands that are likely degradation products of the BirA-tdTomato fusion protein. Arrows denote bands that are likely cross-reactive; they are present independent of BirA-tdTomato expression and are strong in intensity because a long exposure is shown and a large amount of input was loaded. (C) Original scan (3 s) of anti-β-actin Western blots shown in Figure 1—figure supplement 2A. (D) Grayscale scans (left) and brightness and contrast-adjusted scans (right) of Western blots shown in Figure 1—figure supplement 2A. Arrowheads denote bands that likely represent synapsin isoforms. [file elife-83018-fig1-figsupp2-data1.zip › SFigure1_S2_Source_Data_1B-v1.tif]

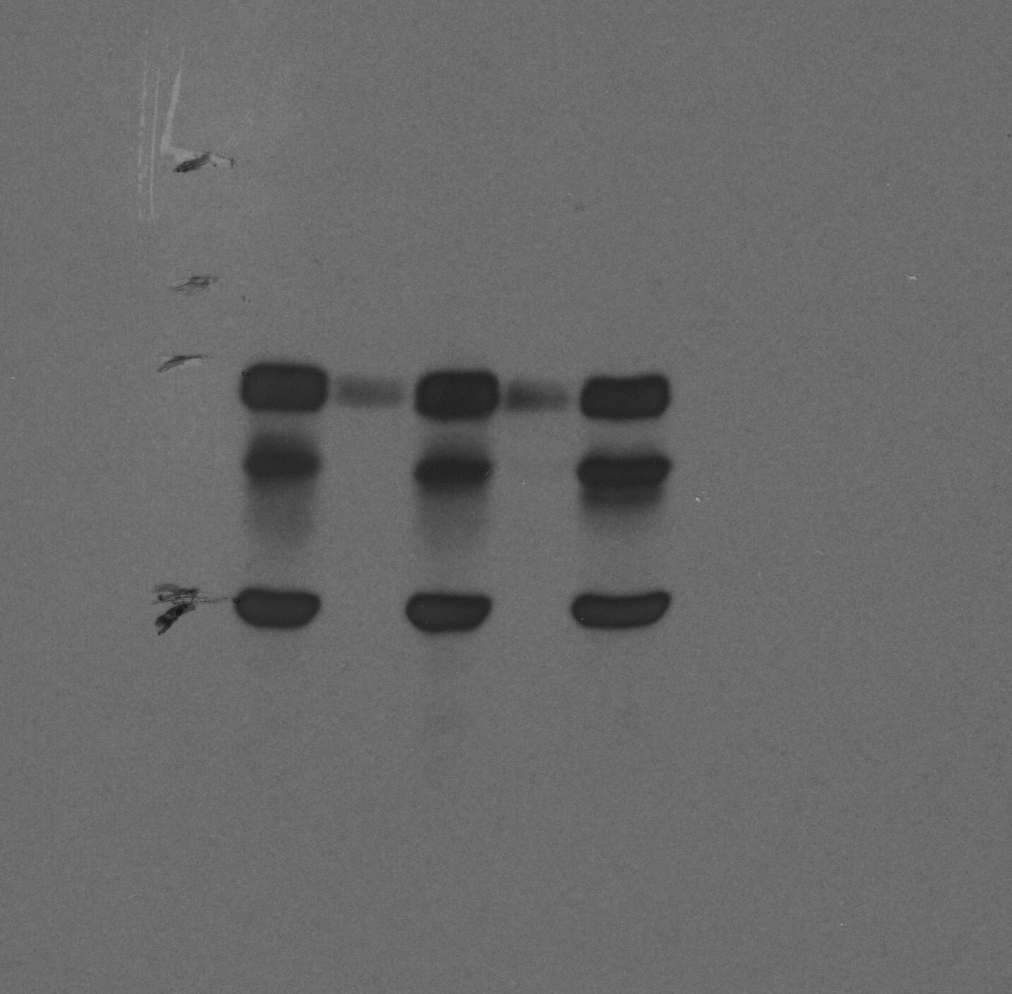

Supplement: Figure 1—figure supplement 2—source data 1. — (A) Original scan (5 min) of anti-RFP Western blots shown in Figure 1—figure supplement 2A. (B) Grayscale scans (left) and brightness and contrast-adjusted scans (right) of Western blots shown in Figure 1—figure supplement 2A. Stars denote bands that are likely degradation products of the BirA-tdTomato fusion protein. Arrows denote bands that are likely cross-reactive; they are present independent of BirA-tdTomato expression and are strong in intensity because a long exposure is shown and a large amount of input was loaded. (C) Original scan (3 s) of anti-β-actin Western blots shown in Figure 1—figure supplement 2A. (D) Grayscale scans (left) and brightness and contrast-adjusted scans (right) of Western blots shown in Figure 1—figure supplement 2A. Arrowheads denote bands that likely represent synapsin isoforms. [file elife-83018-fig1-figsupp2-data1.zip › SFigure1_S2_Source_Data_1C-v1.tif]

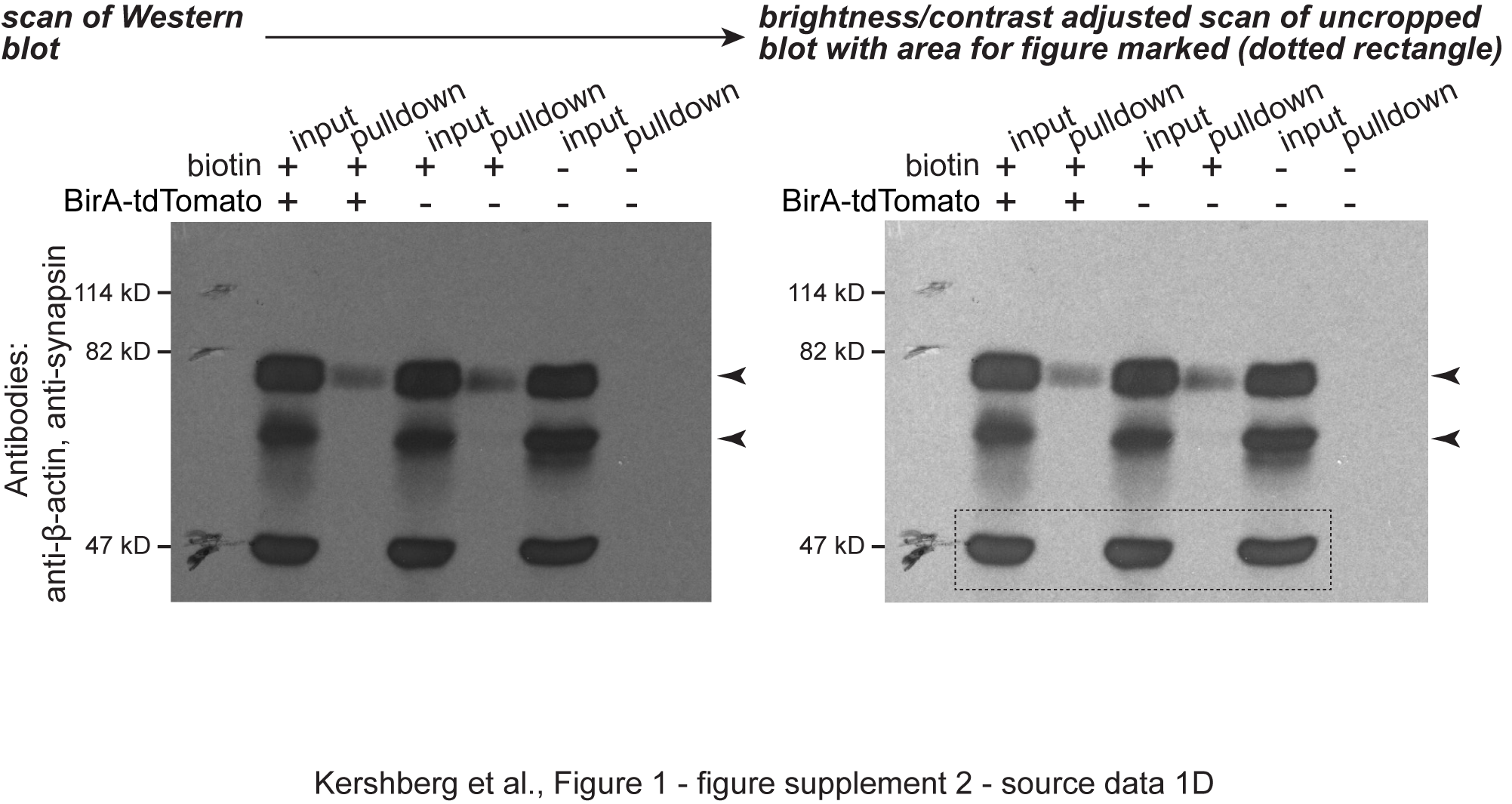

Supplement: Figure 1—figure supplement 2—source data 1. — (A) Original scan (5 min) of anti-RFP Western blots shown in Figure 1—figure supplement 2A. (B) Grayscale scans (left) and brightness and contrast-adjusted scans (right) of Western blots shown in Figure 1—figure supplement 2A. Stars denote bands that are likely degradation products of the BirA-tdTomato fusion protein. Arrows denote bands that are likely cross-reactive; they are present independent of BirA-tdTomato expression and are strong in intensity because a long exposure is shown and a large amount of input was loaded. (C) Original scan (3 s) of anti-β-actin Western blots shown in Figure 1—figure supplement 2A. (D) Grayscale scans (left) and brightness and contrast-adjusted scans (right) of Western blots shown in Figure 1—figure supplement 2A. Arrowheads denote bands that likely represent synapsin isoforms. [file elife-83018-fig1-figsupp2-data1.zip › SFigure1_S2_Source_Data_1D-v1.tif]

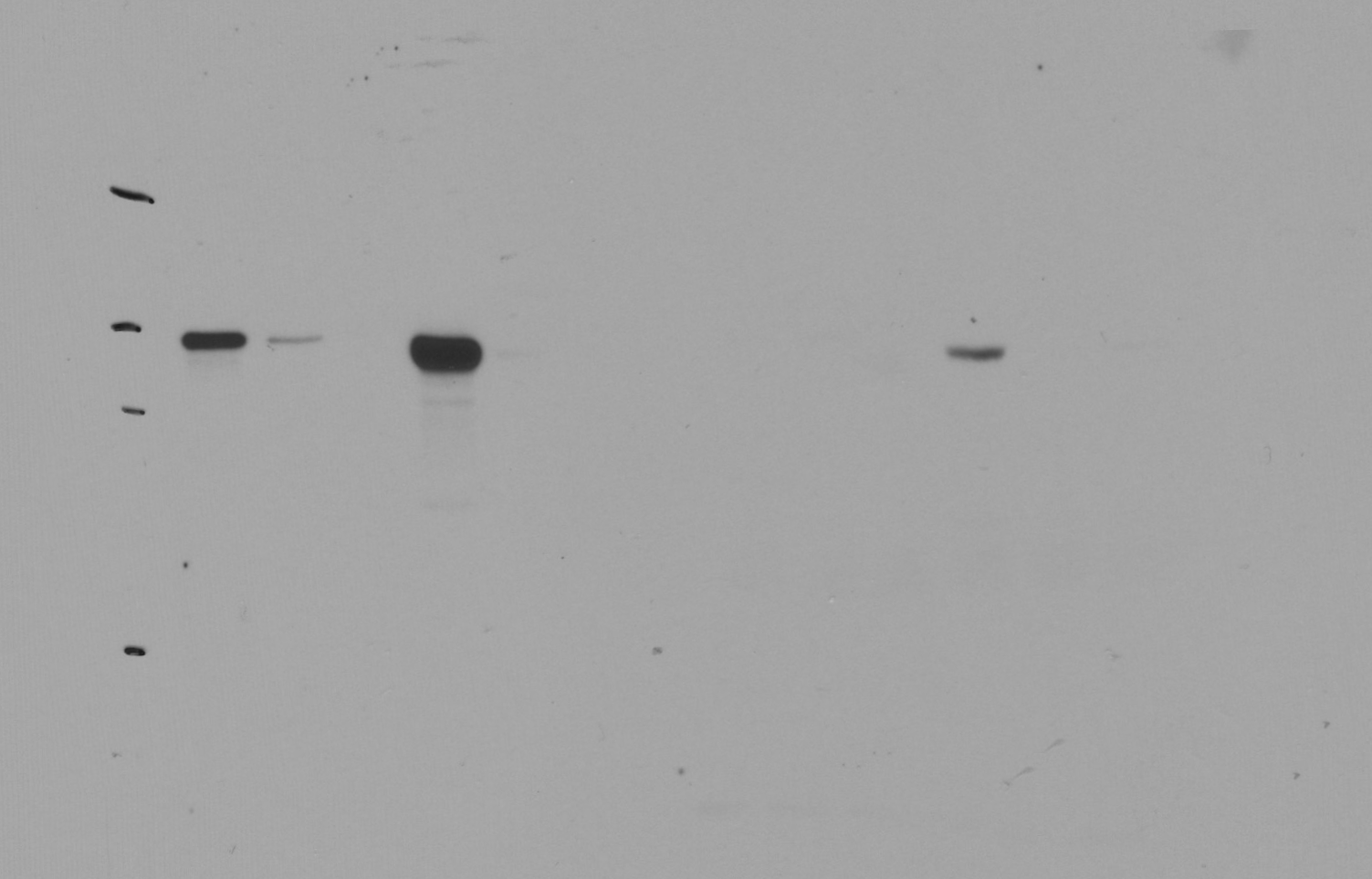

Supplement: Figure 1—figure supplement 2—source data 2. — (A) Original scan (5 min) of anti-HA Western blots shown in Figure 1—figure supplement 2B. (B) Grayscale scans (left) and brightness and contrast-adjusted scans (right) of Western blots shown in Figure 1—figure supplement 2B. [file elife-83018-fig1-figsupp2-data2.zip › SFigure1_S2_Source_Data_2A-v1.tif]

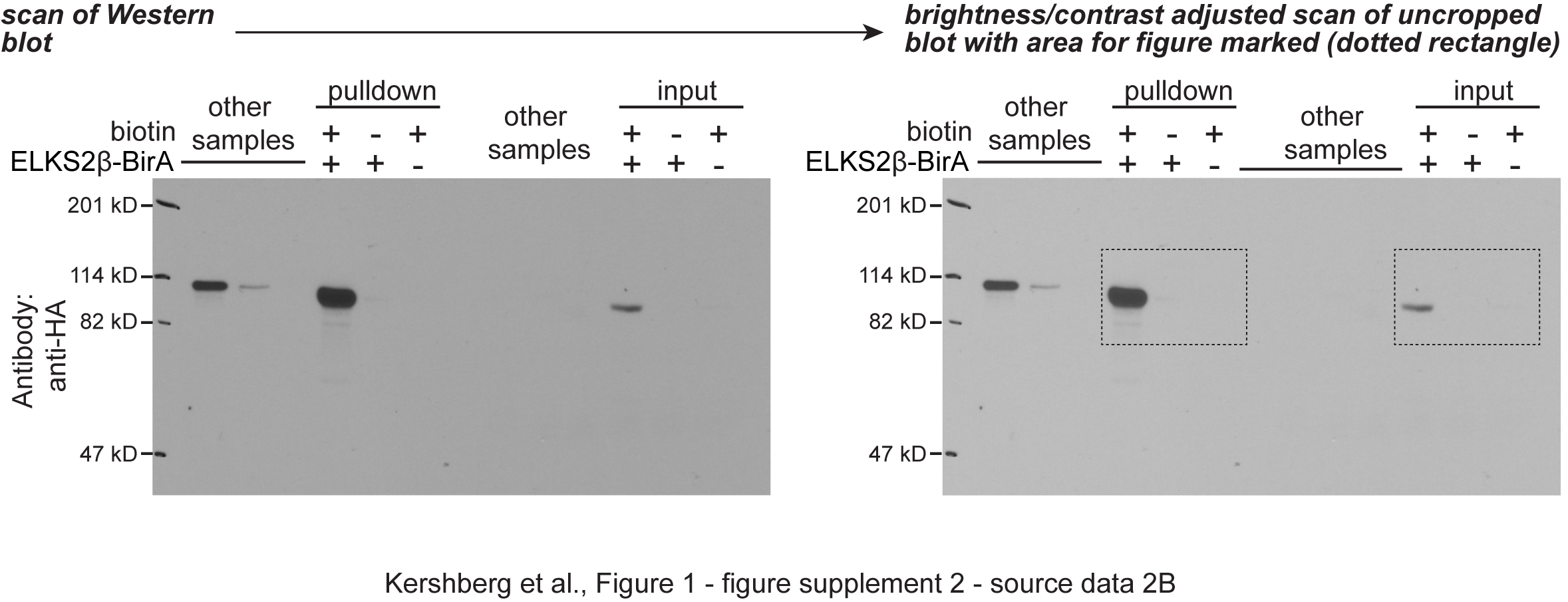

Supplement: Figure 1—figure supplement 2—source data 2. — (A) Original scan (5 min) of anti-HA Western blots shown in Figure 1—figure supplement 2B. (B) Grayscale scans (left) and brightness and contrast-adjusted scans (right) of Western blots shown in Figure 1—figure supplement 2B. [file elife-83018-fig1-figsupp2-data2.zip › SFigure1_S2_Source_Data_2B-v1.tif]
